# Supplementary material for: Prevalence of targeted therapy-related genetic variations in NSCLC and their relationship with clinicopathological characteristics
Source: PLoS One. 2022 Jan 21;17(1):e0262822. doi: 10.1371/journal.pone.0262822 (PMC8782298; doi:10.1371/journal.pone.0262822)
Supplement: S1 Table — (DOCX) [file pone.0262822.s001.docx]

**S1 Table. *Post hoc* analysis results of the association between *ALK* fusion, age, gender, and smoking history of patients and tumor stage**

| Tumor stage | ALK fusion | | | Age | | | Gender | | | Smoking | | |
| --- | --- | --- | --- | --- | --- | --- | --- | --- | --- | --- | --- | --- |
|  | Yes | No | P | < 63 | > 63 | P | Male | Female | P | Smoker | Non-smoker | P |
| I vs.II |  |  |  |  |  |  |  |  |  |  |  |  |
| I | 4 | 120 | 0.072 | 82 | 42 | 0.482 | 51 | 73 | 0.267 | 24 | 100 | 0.06 |
| II | 6 | 50 |  | 34 | 22 |  | 28 | 28 |  | 18 | 38 |  |
| I vs. III |  |  |  |  |  |  |  |  |  |  |  |  |
| I | 4 | 120 | 0.204 | 82 | 42 | <0.001 | 51 | 73 | 0.095 | 24 | 100 | 0.017 |
| III | 5 | 64 |  | 27 | 42 |  | 37 | 32 |  | 24 | 45 |  |
| I vs. IV |  |  |  |  |  |  |  |  |  |  |  |  |
| I | 4 | 120 | 0.493 | 82 | 42 | 0.001 | 51 | 73 | 0.002 | 24 | 100 | 0.001 |
| IV | 3 | 152 |  | 71 | 84 |  | 93 | 62 |  | 59 | 96 |  |
| II vs. III |  |  |  |  |  |  |  |  |  |  |  |  |
| II | 6 | 50 | 0.496 | 34 | 22 | 0.016 | 28 | 28 | 0.687 | 18 | 38 | 0.756 |
| III | 5 | 64 |  | 27 | 42 |  | 37 | 32 |  | 24 | 45 |  |
| II vs. IV |  |  |  |  |  |  |  |  |  |  |  |  |
| II | 6 | 50 | 0.005 | 34 | 22 | 0.056 | 28 | 28 | 0.195 | 18 | 38 | 0.43 |
| IV | 3 | 152 |  | 71 | 84 |  | 93 | 62 |  | 59 | 96 |  |
| III vs. IV |  |  |  |  |  |  |  |  |  |  |  |  |
| III | 5 | 64 | 0.061 | 27 | 42 | 0.352 | 37 | 32 | 0.372 | 24 | 45 | 0.639 |
| IV | 3 | 152 |  | 71 | 84 |  | 93 | 62 |  | 59 | 96 |  |
