# Supplementary material for: Prevalence of targeted therapy-related genetic variations in NSCLC and their relationship with clinicopathological characteristics
Source: PLoS One. 2022 Jan 21;17(1):e0262822. doi: 10.1371/journal.pone.0262822 (PMC8782298; doi:10.1371/journal.pone.0262822)
Supplement: Attachment — Submitted filename: PONE-D-21-30951_reviewer_comments.docx [file pone.0262822.s003.docx]

The study is aimed at assessing the prevalence of sanitizing and resistant mutations among patients with cancer in China. The authors also aimed to understand if there were any associations between genetic alterations and clinicopathological characteristics of patients with cancer. They found that nearly half of the patients (N=404) were eligible for receiving anti-EGFR therapy. Gender could influence the histological type and KRAS mutation frequency among non-smokers.

Title:

The title is indicative of what the study entails.

Abstract:

This should be a standalone section of the manuscript, and therefore, should adequately summarize all sections of the main text. Currently the abstract does not provide enough background or context for the study. Similarly, the methods section of the abstract could be more detailed.

Introduction:

-Although this section provides sufficient background about the incidence of lung cancer globally and in China and also describes the current literature with respect to available therapeutic targets in NSCLC, the rationale for the current study and the current knowledge gap in existing literature have not been clearly highlighted.

-Also, this section does not adequately bring out the originality of the current work and the need for it.

Materials and methods:

-The study design and setting have not been described in this section. The authors have also not mentioned if informed consent was obtained from the participants of this study and whether the study was performed in accordance with ethical standards laid out for studies involving human subjects.

-The inclusion and exclusion criteria used for patient recruitment in the current study have not been described.

-Since, the aim of this study was to investigate the prevalence of genetic variations which indicate either sensitivity or resistance to targeted therapies in NSCLC, it is imperative to provide details about the line of treatment received by the patients. Currently, it is not clear from this section if the patients were treatment-naive or had developed resistance to a previous line of therapy.

-This section currently does not describe the study endpoints.

Results:

This section does not provide information about how many patients were enrolled, how many were excluded, and how many were finally included in the analysis.

Discussion:

The authors have mentioned that 50.7% of NSCLC patients harbored genetic variations sensitive to anti-EGFR therapies (including exon 19 deletion, L858R, L861Q, G719X, and S768I), and that this is higher than the percentage (32.3%) reported by a previous systemic review by Zhang et al.[16]. The authors could try to provide a reason for this considerably higher proportion observed in the current study. Could this be because of the differences in the ethnicity of the two cohorts?

The discussion section does not highlight the novelty of the current research and its possible implications on future research and clinical and/or diagnostic practices.

The conclusions drawn from the results obtained have not been mentioned at the end of the manuscript text.

Tables:

The total cohort size for which the percentages have been denoted needs to be mentioned in the table headers. Moreover, there is considerable duplication of information between table 2 and “biomarker evaluation” section of the results.
